# Appraisal of the Antioxidant Activity, Polyphenolic Content, and Characterization of Selected Himalayan Herbs: Anti-Proliferative Potential in HepG2 Cells

**DOI:** 10.3390/molecules27238629

**Published:** 2022-12-06

**Authors:** Sumaira Yousuf, Shabnam Shabir, Simran Kauts, Tarun Minocha, Ahmad A. Obaid, Anmar A. Khan, Abdulrahman Mujalli, Yahya F. Jamous, Sarah Almaghrabi, Bandar K. Baothman, Ahmed Hjazi, Sandeep K. Singh, Emanuel Vamanu, Mahendra P. Singh

**Affiliations:** 1 School of Bioengineering and Biosciences, Lovely Professional University, Phagwara 144411, India; 2 Department of Zoology, Institute of Sciences, Banaras Hindu University, Varanasi 221005, India; 3 Department of Laboratory Medicine, College of Applied Medical Sciences, Umm Al-Qura University, Makkah 24382, Saudi Arabia; 4 National Center of Vaccines and Bio Processing, King Abdulaziz City for Science and Technology (KACST), Riyadh 12354, Saudi Arabia; 5 Department of Medical Laboratory Sciences, Faculty of Applied Medical Sciences, King Abdulaziz University, Jeddah 21589, Saudi Arabia; 6 Center of Innovations in Personalized Medicine (CIPM), King Abdulaziz University, 21589 Jeddah, Saudi Arabia; 7 Department of Medical Laboratory Technology, Faculty of Applied Medical Sciences in Rabigh, King Abdulaziz University, Jeddah 21589, Saudi Arabia; 8 Department of Medical Laboratory Sciences, College of Applied Medical Sciences, Prince Sattam bin Ab dulaziz University, Al-Kharj 11942, Saudi Arabia; 9 Indian Scientific Education and Technology Foundation, Lucknow 226002, India; 10 Faculty of Biotechnology, University of Agricultural Sciences and Veterinary Medicine, 011464 Bucharest, Romania; 11 Department of Zoology, DDU Gorakhpur University, Gorakhpur 273009, India

**Keywords:** antioxidant activity, Himalayan herbs, HPLC, FT-IR, HepG2, anti-proliferative activity, oxidative stress

## Abstract

Natural antioxidants derived from plants have played a vital role in preventing a wide range of human chronic conditions and provide novel bioactive leads for investigators in pharmacotherapy discovery. This work was designed to examine the ethnopharmacological role of *Urtica dioica (UD)*, *Capsella bursa-pastoris* (CBP), and *Inula racemosa* (IR). The total phenolic and flavonoid contents (TPC and TFC) were illustrated through colorimetric assays, while the antioxidant activity was investigated through DPPH and ABTS assays. The evaluation of phytochemicals by FT-IR of *UD* and CBP revealed high contents of aliphatic amines, while IR showed a major peak for ketones. The antioxidant activity, TPC and TFC were highest in the ethanol extract of UD, followed by CBP, and IR showed the lowest activity. All of the extracts revealed significant antioxidant capacities along a dosage gradient. Through a HPLC analysis at a wavelength of 280 nm, UD leaves demonstrated an intense peak of quercetin, and the peak for rutin was less intense. CBP (whole plant), instead, demonstrated a major yield of rutin, and a peak for quercetin was not observed in CBP. IR (rhizomes) showed both quercetin and rutin. All of the extracts were significantly cytotoxic to HepG2 cells after 48 h with the trend IR > UD > CBP. The outcomes of this study may be effective in the selection of specific plants as realistic sources of the bioactive components that might be useful in the nutraceutical progression and other biomedical efficacies.

## 1. Introduction

In aerobic biological systems, reactive oxygen species (ROS) are consistently produced by a normal cellular metabolism, as well as by external factors, such as environmental contaminants, including pesticides, heavy metals, organic solvents, human carcinogens, and the intentional or non-intentional drug overdose [1]. The phenomenon of the exorbitant free radical production is known as oxidative stress [2]. Free radicals have been linked to the pathophysiology of many diseases, particularly hepatotoxicity, and oxidative stress may be a common mechanism connecting multiple causes for the consequences of diabetes, predominantly vascular dysfunctions, hepatopathy, nephropathy, neuropathy, and retinopathy [3]. The long-term exposure of humans to various pro-oxidant factors, either directly or indirectly, causes severe side effects on human health, as discussed earlier. The contemporary way of life, which includes consuming processed food, being exposed to a variety of chemical toxins and xenobiotics, and lacking physical workouts, is a significant factor in the development of chronic diseases induced by oxidative stress. To restrict this impairment, there is a need for antioxidants that should be incorporated into the daily routine, either through diet or direct administration in the form of supplements [4].

At present, the consumption of synthetic supplements, such as N-acetyl cysteine (NAC), as antioxidants has been shown to promote negative health effects [5]. As alternatives to synthetic compounds, antioxidants derived from natural extracts from plants possess bioactive qualities that bring additional value to the final products. Medicinal herbs play a significant role in the daily regime of humans; approximately 30,000 plant species are used for pharmacology across the globe. In the years ahead, the international beverage and food market is estimated to be worth USD 356.40 billion. Nearly 82% of the world’s population uses herbs and plants as ethnomedicine, with a market value of USD 73 billion. According to epidemiological studies, higher intakes of antioxidant-rich vegetables, fruits, and medicinal herbs are linked with a decreased risk of chronic diseases. Since the high level of free radical production affects apoptosis, cell proliferation, and ion transport, it damages lipids, polypeptides, and DNA and contributes to diseases, including hepatotoxicity [6], cardiovascular disease, carcinogenesis, and neurological disorders [7].

Herbal extracts are multicomponent matrices that can contain thousands of different bioactive components, each of which has a different physiological influence on the human body. The exploration of promising plant-based raw materials, as well as the assessment of their chemical components and pharmacological activities, is especially crucial when designing new medicines for both disease prevention and treatment. As a result, it is critical to use the latest scientific techniques to evaluate the chemical composition of herbal extracts used in traditional and folk medicine to identify which raw materials have the highest anticancer, antioxidant and other ethnopharmacological activities in vitro and in vivo [5]. Antioxidant compounds include nonenzymatic polyphenols consumed through diet and antioxidant enzymes physiologically generated by the body. Small-molecule plant nutraceuticals, such as phenols, caretenoids, and antioxidants containing sulfur, as well as total phenolic antioxidants, make up the majority of nonenzymatic antioxidants (tannins) [8]. The antioxidant properties and phytochemicals obtained from food have demonstrated remarkable chemopreventive effects in a wide range of tumor forms. These phytochemicals also exhibit low or no toxicity against healthy cells, which makes them excellent chemopreventive therapies [9]. For the contemporary study, three herbs from the Northern Himalayan region of India were selected. These medicinal herbs were shortlisted, based on their pharmacological activities, affirmation of their persistent usage, and native accessibility [10].

*Urtica dioica* (UD), *Capsella bursa-pastoris* (CBP), and *Inula racemosa* (IR) are widely used herbs, that are predominantly consumed by people of high altitude as the food itself (UD and CBP) or as medicine (IR). These herbs have been used in the conventional medical system for the prevention of a variety of oxidative stress-related ailments, such as anti-inflammatory [11,12,13,14,15], anti-mutagenic [16], cardiovascular [17], and hepatoprotective [18] diseases, for several decades. The different parts of these plants contain antioxidants that activate the Nrf2-ARE intrinsic antioxidant pathway and maintain the redox homeostasis [19]. Nrf2 is a vital transcription factor that elevates the transcription of various cytoprotective antioxidant genes, such as heme oxygenase-1 (HO-1) and gamma cysteine ligase (γ-GCL), in response to electrophilic, oxidative, and xenobiotic stresses. Nrf2, in its inactive form, resides in the cytosol in association with Kelch-like associating protein-1. In response to oxidative stress, the Nrf2-KEAP1 complex undergoes ubiquitination and translocates to the nucleus, where it starts the transcription of antioxidant enzymes along with enhancer sequences that are antioxidant response elements (AREs) to combat cellular oxidative stress [6].

**Figure 1:**
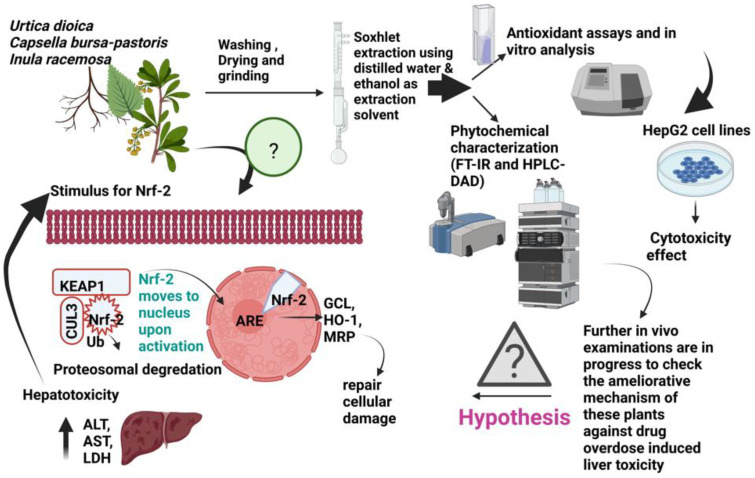
Graphical representation of the antioxidant activity, phenolic, and flavonoid profile cytotoxicity activity in the leaves of UD, whole plants of CBP, and roots of IR in HepG2 cells. Further studies are in continuation on in vivo models and the hypothesis is given on the ameliorative mechanism of these medicinal plants against the drug-induced hepatotoxicity via the Nrf2 signaling pathway [19]. Drug-induced hepatotoxicity is marked by the elevation in liver biomarkers, such as alanine aminotransferase (ALT), aspartate aminotransferase (AST), and lactate dehydrogenase (LDH). Nuclear factor erythroid 2-related factor 2 (Nrf2) is a master transcription factor that enhances the transcription of cytoprotective genes, including heme oxygenase-1 (HO-1), glutamate cysteine ligase (GCL), Mallard reaction products (MRP) and glutathione (GSH) synthesizing and conjugating enzymes, in response to oxidative, electrophilic, and xenobiotic stresses. It is well known that hepatic Nrf2 is activated by drug treatment [21,22].

In the present study, the phytochemical profile of these herbs was judged through the biochemical and analytical methods, such as TFC, TPC, and HPLC-DAD, followed by the evaluation of the cytotoxicity activity of UD, CBP, and IR against HepG2 cells in the quest for an unambiguous differentiation protocol, since the organ protection process is related to cytoprotection. Comparative antiproliferative and antioxidant activity studies of UD, CBP, and IR, distributed in the Kashmir Division of Jammu and Kashmir, India has not yet been conducted. The study was undertaken to examine the cytotoxicity potential through a cell viability test in hepatocellular carcinoma cell lines (HepG2) in order to indicate them as a potential novel conventional medicine to be consumed in the future (Figure 1) [20].

## 2. Results

The outcomes of the evaluations performed to compare the antioxidant and anti-proliferative activities of UD, CBP, and IR, as well as the characterization of secondary metabolites present in these medicinal plants are presented below.

### 2.1. Free Radical Scavenging Capacity of Herbal Fractions

**Figure 2:**
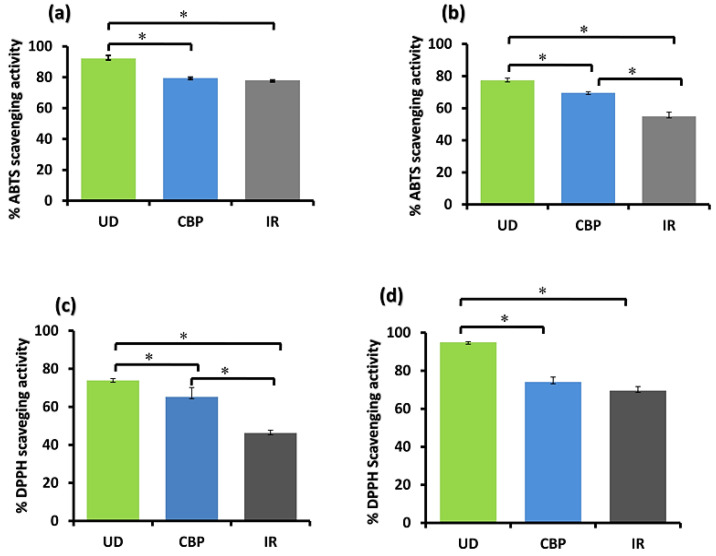
Comparative ABTS and DPPH radical scavenging activity of aqueous (**a**,**c**) and ethanolic extracts (**b**,**d**) of the leaves of *Urtica dioica* (UD), whole plant of *Capsella bursa-pastoris* (CBP), and rhizomes of *Inula racemosa* (IR) at a concentration of 2 mg/mL for DPPH (both AQ and Eth) and ABTS concentration used was 2 mg/mL (AQ) and 0.2 mg/mL (ETH). Data are shown as the mean ± SD for *n* = 3. * *p* < 0.05 was considered statistically significant.

The DPPH test is commonly used for determining the antioxidant ability of plant fractions. Figure 2 presents the findings of the DPPH and ABTS free radical scavenging activities of the extracts. UD (leaves) showed the highest % DPPH radical scavenging activity, with EC_50_ = 0.58 mg/mL (AQ) and 0.23 mg/mL (ETH), followed by CBP (whole plant), demonstrating EC_50_ = 2.82 mg/mL (AQ) and 0.85 mg/mL (ETH), and IR (rhizomes) exhibited the lowest antioxidant activity, with EC_50_ = 2.35 mg/mL (AQ) and 1.06 mg/mL (ETH). The % DPPH scavenging activity of herbs is depicted in Figure 2c,d for the aqueous and ethanolic fractions, respectively.

ABTS is a colored free radical (unstable) engaged to find the antioxidant activities of both hydrophilic and hydrophobic antioxidants of edible extracts. The ABTS radical scavenging potential of the three herbs was in the following order: UD (ETH) (EC_50_ = 0.08 mg/mL) > CBP (ETH) (EC_50_ = 0.09 mg/mL) IR (ETH) > (EC_50_ = 0.17 mg/mL) > UD (AQ) (EC_50_ = 0.41 mg/mL) > IR (AQ) (EC_50_ = 0.79 mg/mL) > CBP (AQ) (EC_50_ = 0.98 mg/mL) (Figure 2a,b) for the aqueous and ethanolic fractions, respectively. The linear regression used to estimate the EC_50_ values has an excellent coefficient of determination (R^2^ ≥ 0.95). The data from the three different tests were statistically analyzed (ANOVA), and it was found that many of the experiments with each sample were statistically equivalent (*p* ≥ 0.05). All of the extracts showed concentration-dependent behavior.

### 2.2. Investigation of Quantification of the TPC and TFC

**Figure 3:**
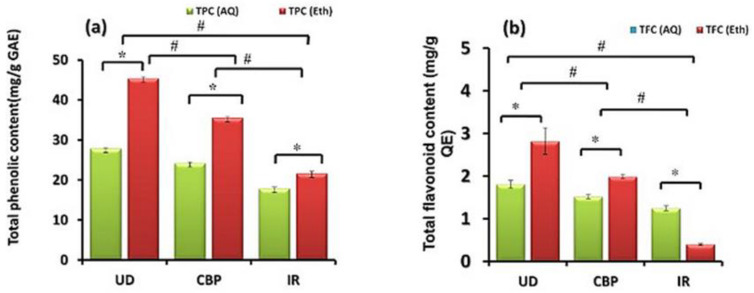
Comparative total phenolic (**a**) and flavonoid (**b**) contents of aqueous (AQ) and ethanolic (Eth) extracts of *Urtica dioica* (UD), *Capsella bursa-pastoris* (CBP), and *Inula racemosa* (IR). Data are shown as the mean ± SD for *n* = 3. * (intragroup) and # (intergroup) *p* < 0.05 were considered statistically significant.

The proportion of the phenolic compounds present in an extract is estimated approximately using the Folin–Ciocalteu reagent. Phosphomolybdic and phosphotungstic acids present in the reagent along with the phenolic compounds present in the extracts, undergo a significant redox process. In this phenolic content assay, various phenolic compounds respond in different ways. In the current research study, the calibration curve of the gallic acid standard acquired for the quantification of phenolic compounds was y = 0.008x − 0.085; R^2^ = 0.999, where x stands for the gallic acid concentration and y for the absorbance. The linear standard curve was obtained between concentrations of 25–100 µg/mL of gallic acid. TPC was observed to be significantly greater in UD than in CBP and IR, as depicted: UD (ETH) (45.39 mg GAE/g), followed by CBP (ETH) (35.52 mg GAE/g), UD (AQ) (27.87 mg GAE/g), CBP (AQ) (24.25 mg GAE/g), IR (ETH) (21.62 mg GAE/g), and IR (AQ) (17.87 mg GAE/g) (Figure 3a). The total flavonoid content (TFC) value was demonstrated as quercetin equivalents as a standard curve (R^2^ = 0.97) obtained by the equation y = 0.116 + 0.097, and the observations were in the following order: UD (ETH) (2.82 mg QE/g), followed by CBP (ETH) (1.99 mg QE/g), UD (AQ) (1.81 mg QE/g), CBP (AQ) (1.52 mg QE/g), IR (AQ) (1.25 mg QE/g), and IR (ETH) (0.40 mg QE/g) (Figure 3b). The assessments of the phenolic and flavonoid concentrations in the extracts may be correlated to their antioxidant activities, since the structural characteristics of the phenolic acids are primarily accountable for the antioxidant potential.

### 2.3. Evaluation of the Correlation Coefficient between the Antioxidant Activity of the Selected Herbal Extracts and Their Phenolic and Flavonoid Contents

According to studies, polyphenols present in herbal extracts are mainly responsible for their antioxidant properties [23]. Therefore, it may be inferred that the TPC and TFC should positively correlate with the DPPH and ABTS scavenging, which is used to assess the antioxidant capabilities. The study also showed the correlation coefficient (R^2^-value) between the phenolic content (UD, CBP, and IR) and the antioxidant potential of the three herbs (Table 1). All samples demonstrated extremely high correlation coefficients between the antioxidant properties and phenolic and flavonoid compositions (aqueous and ethanolic). Therefore, the phenolic components significantly contribute to the antioxidant capabilities of herbs.

### 2.4. Dose Effect Study on the Antioxidant Activity of UD, CBP, and IR

**Figure 4:**
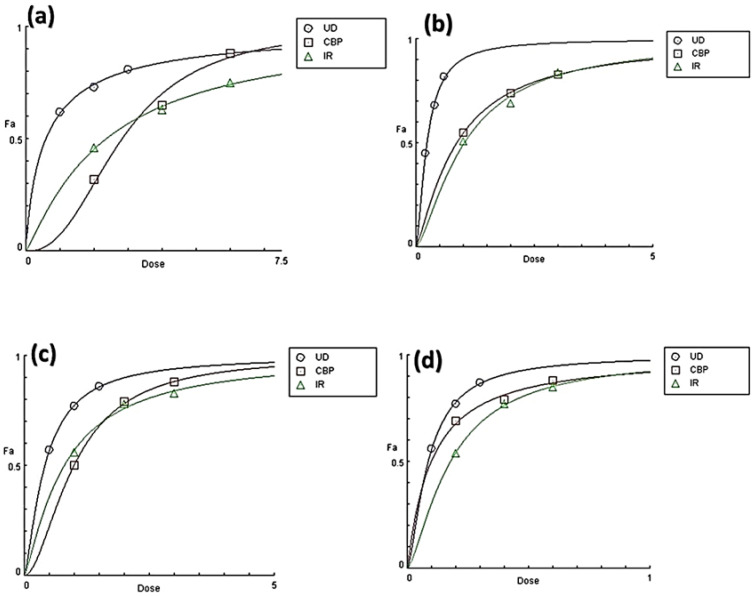
Dose effect curves of aqueous (**a**,**c**) and ethanolic (**b**,**d**) *Urtica dioica* (UD), *Capsella bursa-pastoris* (CBP), and *Inula racemosa* (IR) using DPPH and ABTS radical scavenging models. All three extracts, UD, CBP and IR, showed a dose-dependent behavior. With the increasing concentration, the DPPH and ABTS radical scavenging activities increased significantly. Data are shown as the mean ± SD for *n* = 3. *p* < 0.05 was considered statistically significant.

A dose-response connection is regarded as significant proof of a causal association between exposure and result. Even in the absence of a dose-response association, the possibility of a cause-and-effect relationship cannot be discounted. Dose effect curves of all three herbs, UD, CBP, and IR, were plotted using CompuSyn software (Version 1.0) using three data points (concentrations mg/mL) to obtain EC_50_. All three extracts (both AQ and ETH) demonstrated a dose-dependent behavior. With an increase in the concentration of herbs, the antioxidant activity (Fa) also increased. The highest Fa at 0.50 (EC_50_), Fa at 0.75 (EC_75_), and 0.90 (EC_90_) were shown by UD followed by CBP, and the lowest was shown by IR in both ABTS and DPPH radical scavenging assays, as depicted in Figure 4.

### 2.5. Evaluation of the Functional Groups Present in UD, CBP, and IR via the Fourier Transform Infrared Spectrophotometer (FT-IR) Analysis

FT-IR spectra were used to determine the functional groups of phytochemical constituents found in the herbal extract of UD, CBP, and IR, based on the peak amplitude in the IR radiation area. The functional groups of the individual components were separated according to the ratio of their peaks so when the extract was passed through FT-IR, the analysis affirmed the presence of alkanes, alkyl, and aryl halides, methylene groups, nitro compounds, amino acids, aldehydes, phenols, alcohols, aliphatic primary amines, aromatic compounds, ketones, conjugated alkanes, and halogen compounds.

**Figure 5:**
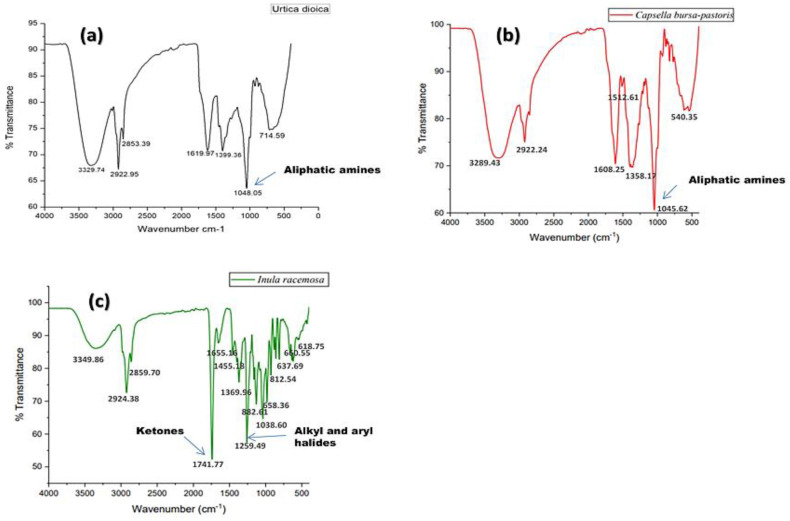
The FT-IR spectra of (**a**) *Urtica dioica* (UD), (**b**) *Capsella bursa-pastoris* (CBP), and (**c**) *Inula racemosa* (IR) with a scan range of 400–4000 cm^−1^.

Equivalent to the highest peak value in the infrared radiation portion of the FT-IR spectrum, the functional group of the bioactive constituents was identified. The spectral arrangement of all herbal fractions was comparable, but the variability in the peak intensity and width, as well as the minor shifts in wavenumber (cm^−1^), was observed. The crude samples of herbs were used for the FT-IR evaluation. The IR spectrum of the crude extract of different herbs, UD, CBP, and IR, is shown in Figure 5, corresponding to Table 2 for UD, Table 3 for CBP, and Table 4 for IR.

The FT-IR evaluation discovered the chemical composition and structure of the ethanolic fractions of UD, CBP, and IR corresponding to the presence of various bioactive compounds, as evidenced by the existence of the intense peaks that have a clear correlation with the therapeutic effects of these herbal fractions on the oxidative stress induced health impairments.

### 2.6. Scrutiny of Phytochemicals by High-Performance Liquid Chromatography-Diode Array Detection (HPLC-DAD)

HPLC was used to accomplish the qualitative analysis and determine the polyphenol content. The ethanolic extracts were subjected to the HPLC analysis of all three samples. As the two main phytochemicals found in natural supplements, quercetin and rutin (HPLC grade) were used as reference standards. Once filtered via 0.45 m Agilent micro filters, the specimens were immersed in vials before being administered to the HPLC. The chromatograms were captured at 280 nm, and the spectral data were collected in the 200–400 nm range.

**Figure 6:**
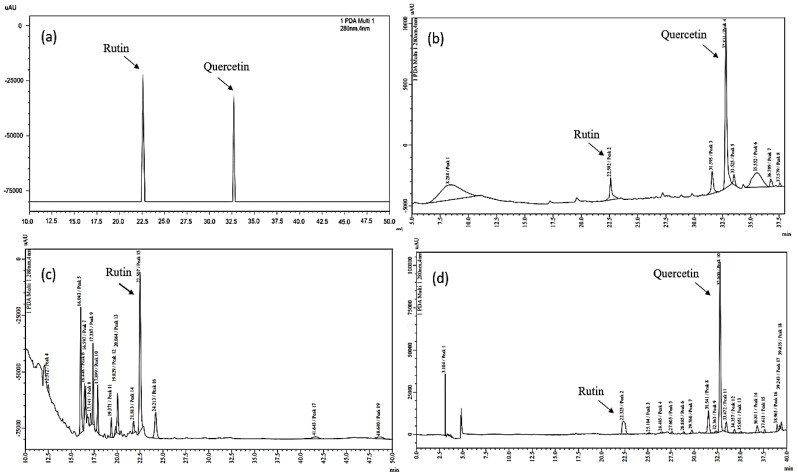
HPLC chromatograms of (**a**) quercetin and rutin as standards, (**b***) U. dioica*, (**c**) *Capsella bursa-pastoris*, and (**d**) *Inula racemosa* were recorded at 280 nm.

The chromatographic profile registered at 280 nm of the ethanolic extract of leaves of UD, CBP, and IR (Figure 6) refers to the phytochemical profile of the abovementioned nutraceuticals. The standard peak illustrated that quercetin showed a peak at a retention time of 32.75 min and rutin at 22.39 min at 280 nm, as demonstrated in Figure 6a. Rutin and quercetin were found to be present in all three selected samples. A peak of quercetin was found in UD and IR at retention times of 32.81 and 32.80 min, respectively, but the peak for quercetin was not found in CBP.

At an injection volume of 50 µL of the ethanolic extract of *Urtica dioica*, it displayed eight peaks illustrated in the chromatogram Figure 6b with a retention time of peak 1 = 8.28, peak 2 = 22.59, peak 3 = 31.59, peak 4 = 32.81, peak 5 = 33.52, peak 6 = 35.55, peak 7 = 36.78, and peak 8 = 37.57 with an area % of 36.15, 5.66, 5.52, 32.87, 1.89, 15.66, 1.68, 0.54 respectively. The major peak i.e., peak 4, corresponded to the presence of quercetin when compared to the retention time of the standards used. The area % of rutin is 5.66% and the quercetin area % is 32.87%. 

The ethanolic fraction of *Capsella bursa-pastoris* (50 µL injection volume) demonstrated a total of 15 peaks, as illustrated in Figure 6c (evaluated as peak 4–peak 19 in the chromatogram) with a retention time of peak 4 = 12.51, peak 5 = 16.04, peak 6 = 16.44, peak 7 = 16.59, peak 8 = 17.14, peak 9 = 17.38, peak 10 = 17.89, peak 11 = 19.37, peak 12 = 19.82, peak 13 = 20.06, peak 14 = 21.81, peak 15 = 22.50, peak 16 = 24.21, peak 17 = 41.64, and peak 19 = 48.64 with an area percentage of 0.99, 19.97, 2.90, 1.85, 1.61, 8.56, 5.86, 2.87, 1.06, 6.69, 2.17, 33.50, 7.68, 2.42 and 1.82, respectively. The major intense peak which is peak 15 represented the presence of rutin with an area % of 33.50% but no peak was obtained that evidenced the existence of quercetin. 

*Inula racemosa* ethanolic fraction (50 µL injection volume) exhibited overall 18 peaks with a retention time of peak 1 = 3.10, peak 2 = 22.32, peak 3 = 25.18, peak 4 = 26.48, peak 5 = 27.60, peak 6 = 28.88, peak 7 = 29.76, peak 8 = 31.54, peak 9 = 32.36, peak 10 = 32.80, peak 11 = 33.47, peak 12 = 34.35, peak 13 = 35.05, peak 14 = 36.81, peak 15 = 37.61, peak 16 = 38.96, peak 17 = 39.24, peak 18 = 39.43 having an area % value of 4.57, 10.97, 0.78, 0.76, 0.89, 0.60, 1.29, 9.64, 0.89, 55.25, 3.95, 1.32, 0.63, 2.95, 1.05, 1.78, 0.86 and 1.77, respectively, shown in Figure 6d. In the *Inula racemosa* ethanolic extract, peaks for both rutin and quercetin were observed at peak 2 and peak 10, respectively. The major peak corresponds to the occurrence of quercetin with an area % of 55.25% and the rutin peak area % observed was 10.97%.

Among all of the polyphenols, quercetin and rutin typically constitutes the major portion of flavonoids present in our daily food. The qualitative analysis of the unknown phytochemicals present in the extracts was benchmarked against the known standard compounds quercetin and rutin, thereby clear evidence of their therapeutic efficacy.

The ubiquitous existence of these important bioactive compounds in selected medicinal plants may contribute to the pharmacological effects of nutraceuticals.

### 2.7. Effect of the Different Herbal Fractions on the HepG2 Carcinoma Cell Proliferation

**Figure 7:**
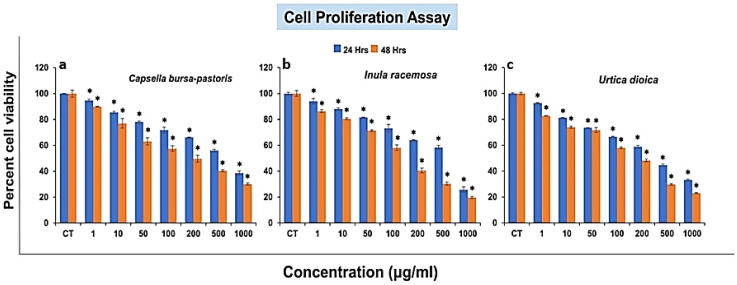
Effects of (**a**) whole plants of *Capsella bursa-pastoris* (CBP), (**b**) rhizomes of *Inula racemosa* (IR) and (**c**) *Urtica dioica* (UD) leaves on the proliferation of a liver cancer cell line (HepG2). HepG2 cells were exposed to various concentrations of drugs, and cell viability was assayed using MTT as a substrate by measuring the absorbance at 570 nm. Data are shown as the mean ± SD for *n* = 3. *p* < 0.05 was considered statistically significant. * Indicates a significant difference between the groups.

**Figure 8:**
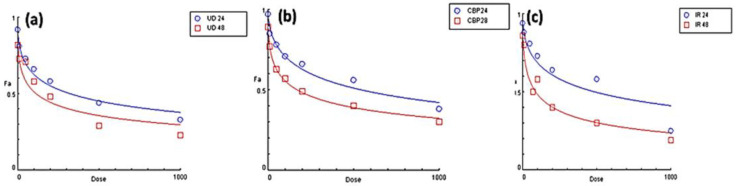
Dose effect curves of (**a**) *Urtica dioica* (UD), (**b**) *Capsella bursa-pastoris* (CBP), and (**c**) *Inula racemosa* (IR) using a MTT cell viability assay using the HepG2 cell lines. All of the extracts showed a dose-dependent response. DRC signifies the effect of the dose on the antiproliferative activity of UD, CBP, and IR. With an increase in the dose, there was a significant decrease in the cell survival. Data are shown as the mean ± SD for *n* = 3. *p* < 0.05 was considered statistically significant.

The cell viability assay results showed that all of the aqueous herbal extracts are potent enough to suppress the proliferation of the HepG2 cancer cells. Aqueous extracts were used to mimic the food ingestion conditions of the normal population. As we increased the concentration from 1–1000 µg/mL, the viability of the HepG2 cells was significantly reduced. The range of viability of the HepG2 cells is demonstrated in Figure 7. IC_50_ values were calculated using CompuSyn Software (version 1.0). *Urtica dioica* (leaves) was confirmed to be the plant extract with the most promising anti-proliferative effect on the Hep-G2 cell line (IC_50_ = 323 μg/mL), followed by the extracts of *Inula racemosa* (roots) (IC_50_ = 488.90 μg/mL), and *Capsella bursa-pastoris* (whole plant) (546.68 μg/mL) at 24 h. The trend for the 48 h for an anti-proliferative activity to the hepatocarcinoma cells was as IR > UD > CBP with IC_50_ values of 87.61 μg/mL, 114.70 μg/mL, and 171.30 μg/mL, respectively. The higher the IC_50_ values, the lower the anti-proliferative activity of the plant extract. All three extracts showed a dose-dependent behavior, as illustrated in Figure 8.

## 3. Discussions

Phytoconstituents present in a variety of medicinal herbs play the main role in the therapeutics of free radical-induced DNA damage, including hepatotoxicity, cardiovascular disorders, neurotoxicity, digestive tract ailments, and even malignancy [24]. The consumption of various pharmaceutical products for the management of these free radical-induced ailments results in a range of adverse effects. Therefore, there is a need for supplements derived from natural sources, such as plants known as antioxidants [25]. The utilization of natural antioxidants is considered to be safe, as they have fewer mutagenic effects and are safe for most of the population. Spices and herbs are among the major imperative targets to explore for conventional antioxidants by safety. The therapeutic efficacy of herbal medicines is determined by the chemical composition of the active phytochemical constituents and the pharmacological activity they have on the oxidants of the body [26]. The notion that most medicinal herbs have a complicated chemical makeup that ranges from two to three components to tens or even hundreds underlie their diverse pharmacodynamic effects [27].

For the present study, the three medicinal plants consumed by the local habitants from the Northern Himalayan regions of India, UD, CBP, and IR were selected for the comparative analysis of antioxidant activity, phenolic content, and anti-proliferative efficacy. These herbs were shortlisted, based on the basis of their ethnopharmacological activities. Previous investigations have been performed to analyze the phytochemical profile of these herbs through analytical methods, such as FT-IR, NMR spectroscopy, and HPLC-DAD [14,28,29,30,31].

However, to the best of our knowledge, there is no comparative analysis of these herbs in the literature, and in the current study, we attempt to provide a comparative account of all three herbs via analytical and biochemical parameters, including FT-IR, HPLC-DAD, phytochemical properties, antioxidant profile, and antiproliferative activity. The aqueous and ethanolic extracts of UD, CBP, and IR individually were determined for the analysis of the antioxidant activity at different concentrations against DPPH and ABTS and the quantification of TFC and TPC was executed using quercetin and gallic acid as reference compounds, respectively.

Among all the herbal extracts, in the DPPH radical scavenging assay, UD manifested the lowest EC_50_, followed by CBP, and IR exhibited the lowest antioxidant activity, with the highest EC_50_ value. The % DPPH scavenging activity of herbs is depicted in Figure 2. The same trend was followed in the ABTS radical scavenging assay. For both aqueous and ethanolic extracts, UD demonstrated the highest DPPH and ABTS radical scavenging activity, followed by CBP, and IR showed the lowest scavenging activity. In comparison to a previous study performed by Fattahi et al. (2014) [32], our results demonstrated the presence of a higher TPC, TFC, and antioxidant activity in UD. The amount of TPC and TFC in the ethanolic fractions was much higher than in the distilled water extracts, showing that the alcoholic fraction of UD showed a higher TPC and TFC, compared to other extracts. A similar trend was demonstrated by Bhatt and Parajuli (2017), in which a methanolic extract of UD showed a greater antioxidant activity, as compared to other extracts [33]. Ethanolic extracts exhibited the highest antioxidant activity through ABTS and DPPH, compared with aqueous extracts.

A previous study demonstrated that UD is considered to have a greater antioxidant activity than some other medicinal plants that are due to the presence of its major compound quercetin, isorhamnetin, and kaempferol [34]. Ethanolic extracts of all three herbs demonstrated the highest scavenging activity, compared to aqueous extracts. The discrepancy between the ROS/RNS scavenging activity and the phenolic content obtained and previous research, may be due to the variations in pre- and post-harvest conditions (e.g., soil conditions, organic manure type, weather conditions, and genetic differences), which could play a significant role in the nutritional accumulation variability [35]. For instance, different concentrations of nutrients were found in almost the same species of stinging nettle grown in different agro ecological conditions [36].

Based on the analysis through spectrophotometric assays, the determined concentration of phenolic substances differed substantially among the plant fractions and techniques used for extraction. Certainly, the composition of extraction techniques influences the concentration and production of phenolic extracts from plant sources. The extraction competence of the phenolic substances is affected by different solvent types with varied polarities. Phenolic substances are frequently extracted in greater quantities in highly polar solvents. However, the solubility of the phenolic acids is determined not only by solvent polarity, but also by the chemical character of the phenolic substances.

For all three extracts, UD, CBP, and IR, higher quantities of phytochemicals were found in ethanolic extracts than in aqueous extracts. For phenolic extracts from herbs, an efficacy order of the solvent forms methanol > aqueous > ethanol > acetone, was obtained [37]. However, the TPC and TFC were found to be greater in UD trailed by CBP, and the lowest content was shown in IR, demonstrating the lowest phenolic and flavonoid contents. The current investigation demonstrated higher yields of TPC and TFC in ethanolic extracts than in aqueous extracts. This could be due to the solvent type used for extraction, as ethanol was found to be best for the extraction of flavonoids and phenols [38]. Specifically, alcoholic solvents were substantially more productive than aqueous solvents at recovering tannins, which was likely due to the polar character of ethanol, which could give hydroxyl ions and make it convenient to interact with polar functional groups of tannins. The aqueous extraction, moreover, was shown to be highly beneficial in terms of the total anthocyanin and 3-deoxyanthocyanidins. TFC, in the case of these variances, could be attributed to the phenolic components’ differing physicochemical characteristics. In the case of IR, TFC in the aqueous extract was found to be greater than that in the ethanolic extracts. Mohan and Gupta, 2017 showed that the roots of IR had the highest TFC in aqueous extracts, compared to other extracts, such as hydro alcoholic and ethanolic extracts [39].

Most of the earlier studies confirmed that the amount of the phenolic and flavonoid content is directly proportional to the antioxidant capability of plant extracts. This was confirmed by the correlation coefficient analysis between TPC/TFC-DPPH and TPC/TFC-ABTS. Almost all of the extracts showed a strong positive correlation between TPC/TFC and the % scavenging activity of the herbal extracts. The quantities of phenolic components obtained from several extraction techniques and herbal extracts in this study were largely consistent with previous studies. Various temperatures, extraction durations, sample-solvent ratios, agitating protocols, and numbers of replicate extractions, can all affect the efficiency of the extraction. Differences in the genetic makeup (genotypes) of plants may also add to the differences in the phenolic content between the same plant species with a different genetic makeup [40].

The FT-IR method was used to identify the type of inorganic and organic compounds found in plants. The experiments were run on drying and low-temperature material from various plant components. The FT-IR spectrum was determined in the range of 400–4000 cm^−1^. The FT-IR analysis of UD, CBP, and IR, as shown in Figure 5a–c, respectively, exhibited the presence of phenols and alcohols in the range of 3000–3500 cm^−1^, alkanes and aldehydes (2500–3000 cm^−1^), aromatic compounds, nitro compounds and methylene groups in the range of 1100–2000 cm^−1^), aliphatic amines, alkenes, and alkyl and aryl halides (500–1100 cm^−1^). The FT-IR peaks of UD, CBP, and IR in the present study were successfully correlated with the previous literature [41,42,43]. All of the peaks obtained were similar to those of the previous studies mentioned earlier. In the case of IR, some additional peaks were obtained, compared to UD and CBP, such as the peak for ketones at 1741 cm^−1^_._ Peaks for alkenes and conjugated alkanes were obtained in IR at wavenumbers 658 cm^−1^, 660 cm^−1,^ and 1655 cm^−1^, respectively, which were not seen in UD and CBP. The major ketone present in IR is sesquiterpene lactones, such as alantolactone and isoalantolactone. These are the major phytochemicals present in IR roots that are responsible for various pharmacological activities [44].

The characterization of the phytochemicals present in plant extracts provides researchers with a thorough perspective of their pharmacological usage. The preliminary phytochemical investigation through the HPLC-DAD analysis confirmed the presence of various bioactive compounds in ethanolic fractions of all three plant species (UD, CBP, and IR). The chromatogram recorded at 280 nm demonstrated the highest peak with injection volumes 50 µL, as illustrated in Figure 6a–d for standard, UD, CBP, and IR, respectively. The identification of standard compounds in herbal fractions was determined, according to the retention time acquired from the standard run at comparable conditions. The two major peaks were segregated and identified with an identical value between the standard retention time and herbal extracts.

The chromatographic profile registered at 280 nm of the ethanolic extract of leaves of UD refers to the leaf phenolic composition. The HPLC results of the ethanolic extract of UD demonstrated the presence of various flavonoid compounds, such as rutin and quercetin. CBP showed peaks that proved the presence of rutin, but quercetin was absent in CBP. The earlier literature reported the presence of quercetin and rutin as major flavonoids in UD, along with some other phenolic compounds, such as caffeic acid, chlorogenic acid, and rosmarinic acid. Due to the presence of quercetin and hydroxycinnamic acid, UD possesses an antimicrobial potential, and rutin administration alleviates the hippocampal neuronal damage in rodents [45,46]. The methanolic extract of UD examined by Joshi and Mukhija (2015) was rich in quinic acid [47], kaempferol-3-O-rutinoside, palmitic acid, arginine, and β-sitosterol, described earlier by Grosso et al. (2011) [48]. In the CBP ethanolic herbal fraction, rutin was observed with a peak area % of 33.50% and a peak for quercetin was not witnessed, but earlier observations identified the existence of the glycosides of quercetin, kaempferol, chrysoeriol, and isorhamnetin. Isorhamnetin-O-rutinoside and Chrysoeriol-O-glucoside were observed in this plant for the first time and it was concluded that the presence of these phytochemicals had a strong correlation with the antioxidant activity of this plant against the DPPH-free radical [49].

The difference in the outcomes could be due to the demographic differences of the plants, and the difference in the solvent and extraction procedures used, as the solvent used for the extraction could be highly responsible for the amount of phenolic content in the extract obtained. IR illustrated the presence of both peaks corresponding to quercetin with a peak area % of 55.253% and the rutin peak area % observed was 10.97%. All three extracts demonstrated different phytochemical profiles. Rutin was found in all three extracts, but quercetin was found to be absent in CBP. An intense peak of quercetin was obtained in UD and IR, and an intense peak for rutin was found in CBP. The peak for rutin was not vigorous in UD and IR. Previous studies also showed the presence of similar compounds in UD leaves, and a slight difference was observed, due to the climatic differences in the habitat of the plant [36].

The global burden of liver cancer is continuously increasing daily, affecting the world population [50]. A wide array of agents, including phytochemicals, is prevalent in the treatment of this chronic disease, but the survival rates are still unsatisfactory [51]. Therefore, the current study was designed to investigate the anticancer potential of extracts UD, CBP, and IR against liver cancer cells, and it was observed that each drug showed a strong anticancer potential, significantly inhibiting the proliferation of the HepG2 cells in a dose- and time-dependent fashion. 

Previous studies showed that the methanolic extract of UD showed an inhibitory activity on the HepG2 cells at an IC_50_ of 420 µg/mL after 48 h. Our results exhibited an IC_50_ of 114 µg/mL at the time interval of 48 h, which indicates the higher cytotoxicity of UD in the current study [52]. The difference in the anticancer activity could be due to the different solvents used, which could lead to a difference in the yields of the phytochemicals with anticancer activities and their ability to be bioavailable readily [53]. A comparative analysis showed that after 48 h, IR showed the highest anticancer activity, followed by UD, and CBP demonstrated the lowest antiproliferative activity in the HepG2 cell lines. The reason could be that the IR showed the intense peak of ketones corresponding to sesquiterpene lactones through the FT-IR and demonstrated the presence of both quercetin and rutin via HPLC-DAD. Sesquiterpene lactones illustrate an anticancer potential through various alterations in the homeostasis of the cells [54] and the effect of these on the different types of signaling mechanisms, primarily the Nrf-2 signaling pathway. These pathways lead to a decrease in the expression of various factors responsible for abnormal cell cycles, the elevation in apoptotic factors, and a decrease in antiapoptotic, proliferative, and carcinoma cell invasive factors [55]. In the future, we will examine the ameliorative effect of these nutraceuticals on drug overdose-induced hepatotoxicity and nephrotoxicity in a mammalian model.

## 4. Materials and Methods

### 4.1. Chemicals and Reagents

Aluminum chloride (AlCl_3_), Folin–Ciocalteu reagent (FC), and potassium persulfate (K_2_S_2_O_8_) were procured from Loba Chemie Pvt. Ltd. Colaba Mumbai, India, 2,2′-azinobis (3-ethylbenzothiazoline-6-sulfonic acid) (ABTS), gallic acid (C_6_H_2_(OH)_3_CO_2_H), 1,-1-diphenyl-2-picrylhydrazyl (DPPH), hydrochloric acid (HCl), acetate buffer, phosphate-buffered saline (PBS), sodium carbonate (Na_2_CO_3_), quercetin (C_15_H_10_O_7_), rutin (C_27_H_30_O_16_), ethanol (C_2_H_5_OH), fetal bovine serum (FBS), trichloroacetic acid (TCA) (C_12_HCl_3_O_2_), acetic acid (CH_3_COOH), and Trizma base (C_4_H_11_NO_3_) were obtained from Hi-Media (India). For HPLC, analytical grade methanol was used Loba Chemie Pvt. Ltd. Colaba Mumbai, India. Water was deionized using a Milli-Q system. A colorimetric analysis was performed using an advanced microprocessor UV–VIS single beam spectrophotometer L1-295 (Panomex New Delhi, India).

### 4.2. Origin, Collection, and Authentication of the Plant Materials Used

**Figure 9:**
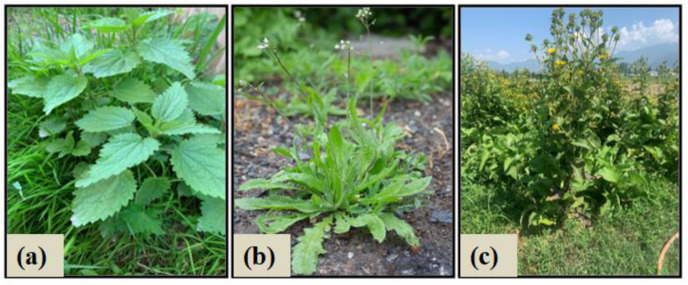
Illustration of the medicinal plants used for the study (**a**) *Urtica dioica* (Family: Urticaceae) (**b**) *Capsella bursa-pastoris* (Family: Brassicaceae) (**c**) *Inula racemosa* (Family: Asteraceae) commonly known as stinging nettle, shepherd’s purse and pushkarmool, respectively.

Young leaves of *Urtica dioica* (Figure 9a) and whole plants of *Capsella bursa-pastoris* (Figure 9b) were harvested from the apple orchards and grasslands of Sopore town of District Baramulla of Jammu and Kashmir, India, in April. These two herbs are wild-grown medicinal plants found in almost all terrestrial parts of North Kashmir, Jammu and Kashmir, India. *Inula racemosa* is found in parts of the Kashmir Himalayas in India. It is also cultivated at Sher-e-Kashmir University of Agricultural Sciences and Technology (SKUAST) (Figure 9c), and the sample for the current investigation was collected from the Ayush Department of SKAUST-K Wadura, Sopore, Jammu and Kashmir, India, in May. All the herbariums were prepared according to the botanical rules and were submitted to KASH Herbarium, University of Kashmir. Plant materials were validated and authenticated by a taxonomist at the University of Kashmir Jammu and Kashmir, India as *Urtica dioica* (6021-KASH), *Capsella bursa-pastoris* (6023-KASH), and *Inula racemosa* (6022-KASH). All the plants originate in the Himalayan regions of North India, including Himachal Pradesh and Jammu, and Kashmir [55].

*Urtica dioica* leaves were removed from the stems and washed under tap water to clean the leaves. These leaves were kept in shade for drying, then ground to form a fine powder, and stored for future analysis. The whole plant of *Capsella bursa-pastoris* was dug from the surface of the ground, along with roots using a knife. The collected plants were rinsed using tap water and then shade-dried. The dried plant samples were ground and the powder was stored in airtight jars for further investigation. Fresh roots of *Inula racemosa* were excavated and the stem of the plant was removed. The roots were thoroughly washed to remove the earth’s remains and debris. The roots were then divested and chopped into small bits and were allowed to shade dry. The dried sample of the roots was blended to form a fine powder using an electric blender, and stored in airtight containers. Ten percent aqueous (AQ) and ethanolic (ETH) extracts (20 g powder in 200 mL extraction solvent) were prepared using the Soxhlet method for 24 h [56]. The solvent of the filtrates was evaporated to obtain a crude extract using a rotary evaporator and refrigerated at 4 °C for further examination. The two different solvents were used to compare the efficacy of the extraction solvents [57].

### 4.3. Antioxidant Activity through the ABTS and DPPH Radical-Scavenging Assays

The antioxidant activity of the plant extracts was determined by DPPH and ABTS antioxidant assays, following the protocol previously mentioned by Mensor et al. (2001) and Re et al. (1999), respectively [58,59].

#### 4.3.1. DPPH Radical Scavenging Activity of the Plant Extracts

A fresh stock of DPPH (11 mg) was dissolved in 50 mL of methanol for the spectrophotometric observations. The DPPH stock solution was diluted with 50% methanol and the optical density/absorbance (OD/Abs) was maintained between 0.8–1. Various plant extract concentrations were administered to a 2 mL DPPH stock solution in sterilized test tubes. Following the incubation for 30 min in a water bath at 37 °C, the OD was analyzed at 520 nm using a spectrophotometer (Shimadzu UV-1601, Japan) [58]. The untreated DPPH stock was used as a control. Triplicate readings were measured. The scavenging activity of the herbal fractions was calculated by:

% scavenging activity = (Absorbance of control − Absorbance of test)/Absorbance of control × 100(1)

#### 4.3.2. ABTS Radical Scavenging Activity of the Plant Extracts

The radical cation decolorization analyte 2, 2′-azinobis (3-ethylbenzothiazoline-6-sulfonic acid) has also been used to evaluate the efficacy of plant samples to quench reactive oxygen species that is relied on for the lessening of ABTS (radicals) by antioxidants in herbal extracts. Firstly, the stock solution of ABTS (36 mg in 10 mL methanol) was mixed with potassium persulfate (57 mg in 10 mL of methanol) in a 1:1 ratio, to form a radical cation. The incubation of this stock mixture was carried out in dark conditions for 14–16 h at room temperature. The ABTS solution was diluted with 50% methanol to obtain an OD of 0.8–1. Different concentrations were added to every 2 mL of the ABTS stock solution. Following the incubation for 30 min in a water bath at 37 °C, the OD was taken at 745 nm [59]. The percentage of the scavenging activity was calculated by the Formula (1).

### 4.4. Estimation of the Total Phenolic Content (TPC)

The total phenolic content in aqueous and ethanolic fractions of UD, CBP, and IR was estimated using the Folin–Ciocalteu reagent protocol, mentioned by Singleton et al. (1999) [60]. The Folin–Ciocalteu reagent 5 mL (0.2 N) was added, preceded by the requisite volumes of herbal fractions, and assorted. Five min later, 4 mL of 7.5% sodium carbonate (Na_2_CO_3_) was administered and thoroughly mixed. The samples were kept in the dark for 120 min at 37 °C. The optical density was then determined at 760 nm. The TPC was expressed as mg gallic acid equivalents (GAE) per g.

### 4.5. Quantification of the Total Flavonoid Content (TFC)

The total flavonoid content of the selected medicinal herbs was calculated using a colorimetric test (aluminum chloride), as previously described by Zhishen et al. (1999) [61]. By dissolving the required amount of quercetin in distilled water, a standard solution of quercetin was prepared. Then, 1.50 mL of methanol was mixed with 0.50 mL of herbs, and both aqueous and ethanolic extracts and incubated for 5 min. Following incubation, a 10% aluminum chloride solution was equipped by dissolving aluminum chloride in distilled water; 4 ml of this solution was added to the extracts and left for 5 min. To the above mixtures, 100 mL of potassium acetate solution (made by dissolving 9.80 g potassium acetate in 100 mL distilled water) was incorporated. Then, after 30 min, 2.90 mL of distilled water is added and the optical density at 415 nm is measured.

### 4.6. Fourier Transform Infrared Spectroscopy (FT-IR)

The active functional groups in the extract were distinguished using FT-IR spectroscopy. The FT-IR analysis provides information about a molecule’s structure, chemical bonds, and functional groups of the extract, which is often acquired from its spectral region. The absorbance of the wavelength of light indicates the chemical bond which is observed in the spectrum obtained.

The FT-IR spectrophotometric analysis of *U. dioica*, *Capsella bursa-pastoris*, and *Inula racemosa* was performed for scrutiny using Shimadzu IR affinity Japan. All ethanolic extracts of the selected samples (10 mg) were embedded in KBr pellet (100 mg), in order to form translucent sample discs and mounted into ATR-FTIR spectra of 350–4000 cm^−1^ in the infrared area of the spectrum at room temperature. The PerkinElmer spectrum IR has been used to analyze the functional groups in the ethanolic extracts [62].

### 4.7. HPLC-DAD Analysis

#### Sample Processing

The HPLC-DAD analytical procedure was carried out to segregate the diverse natural ingredients from the extracts of *Urtica dioica*, *Capsella bursa-pastoris*, and *Inula racemosa*. The ethanolic extracts were used in the HPLC analysis of the plant samples. A rotary evaporator at 50 °C was used to dry up the ethanol from the sample solution until a creamy concentrate was collected. Crude extracts were transferred in micro centrifuge tubes and stored at 4 °C until further analysis. Ethanolic fractions were redissolved in 1ml of HPLC grade methanol and were centrifuged for five minutes at 10,000 rpm throughout the sample processing. The herbal fractions were filtered via 0.45 µm Agilent micro filters, placed in vials, and then run through HPLC.

The extracts were evaluated using a previously reported procedure on an analytical HPLC unit Gilson Illinois, USA [63]. To mitigate the pressure variations caused by the mixing of methanol (MeOH) in water, four pumps (pump model LC-20AD with a low-pressure gradient mode) (A, B, C, and D) were used to mix the mobile phase. To increase the peak resolution, formic acid (5%) was appended to both methanol and water before preparing the mentioned mobile phases: 95% water + 5% methanol (A); 88% water + 12% methanol (B); 20% water + 80% methanol (C); and methanol (D). The solvents used were HPLC grade. The elution began with 100% A and remained isocratic for 5 min. Then, after 10 min, a gradient has been used to reach 100% B, which was then held isocratic for 3 min. From 13 to 35 min, a linear gradient was used to achieve 75% B and 25% C, then 50% B and 50% C at 50 min, and 100% C at 52 min, before remaining isocratic until 57 min. Then, the column was washed with 100% D. The spectral data were obtained in the 200–400 nm range, and the chromatograms were documented at 280 nm. The qualitative analysis of the unknown phytochemicals present in the extracts was benchmarked against the known standard compounds quercetin and rutin (HPLC grade), thereby summarizing clear evidence of their therapeutic efficacy. The spectral data were obtained in the range of 200–400 nm, and the chromatograms were recorded at 280 nm.

A diode-array detector/photodiode-array detector (PDA) was used for the detection of the spectral data. The LC stop time: 57.01 min, PDA detector name: PDA, PDA sampling frequency: 1.5625 Hz, PDA start time: 0.00 min, PDA end time: 57.01 min, PDA time constant: 0.640 sec and the column oven (CTO-10ASvp) temperature was 30 °C. The autosampler model: SIL-20AC, sample rack: Rack 1.5 mL 105 vials, rinsing volume: 500 µL, needle stroke: 52 mm, control vial needle stroke: 52 mm, rinsing speed: 35 µL/s, sampling speed: 15 µL/s, purge time: 25.0 min, rinse dip time: 0 s, cooler temperature: 15 °C.

### 4.8. Anti-Proliferative Efficacy Analysis Using the MTT Assay

To study the cytotoxicity of the extracts selected on the cell survival, the HepG2 cells were incubated using different concentrations of herbal extracts (1 µg, 10 µg, 50 µg, 100 µg, 200 µg, 500 µg, and 1000 µg) of all three plants. The cytotoxicity of *Capsella bursa-pastoris*, *Inula racemosa,* and *Urtica dioica* was assessed in the liver cancer HepG2 cell line, as per the method described earlier [64]. The HepG2 cells were grown-up in 75 cm^2^ flasks in DMEM (Gibco Dulbecco’s Modified Eagle Medium), augmented with 100 U/mL penicillin, 5% fetal bovine serum (FBS), and 100 mg/mL streptomycin under standard conditions (humid environment, 5% CO_2_ and 37 °C) and the cells were sub-cultured after every 4 days, and the growth media was renewed after every 2 days. First, the cells were adhered overnight in a 96-well microtiter plate, at a density of 2.5 × 103 cells per well, followed by treatment with the desired concentrations of the abovementioned drugs for 24 and 48 h. Following the stipulated time, 10 μL of MTT (5 mg/mL stock) was added to each well and allowed to incubate for 2 h. Thereafter, 100 μL of DMSO was added into each well to dissolve the intracellular formazan, and the absorbance was read at 570 nm using an ELISA plate reader. The control contained the culture media and cells only excluding the plant extracts to be analyzed.

### 4.9. EC_50_ Illustration of the Extracts of the Medicinal Plants Used

EC_50_ is an effective dosage at which 50% of the free radicals are consumed. EC_50_ is a major parameter used to demonstrate the antioxidant capacity of the substances. The EC_50_ could be illustrated by the interpolation of the data from a suitable curve or by a nonlinear regression of the data, by using various models [11].

### 4.10. Statistical Analysis

All data points were articulated in triplicate and with three different tests. The data are given as the mean ± standard deviation (SD) using Microsoft Excel 2007. The EC_50_ values were obtained using CompuSyn Software (version 1.0). The statistical analysis between the two groups was performed using a one-way ANOVA (analysis of variance) and Tukey’s test with SPSS (Statistical Package for Social Sciences) software (version 18). The probability standards of *p* < 0.05 were considered statistically significant [65,66].

## 5. Conclusions

The reactive oxygen species-induced serious health conditions, such as liver disease, kills approximately 2 million individuals globally each year, 1 million from cirrhosis complications and 1 million from alcoholic liver disease and hepatic cancer. Natural antioxidants are considered to be the best therapies for combating free radical-induced ailments. From the current study, it was concluded that the antioxidant and anticancer potential of *Urtica dioica*, *Capsella bursa-pastoris*, and *Inula racemosa* has statistically varied (*p* < 0.001) antioxidant and antiproliferative activities. This study has provided preliminary information to investigate the chemical profile of UD, CBP, and IR using the FT-IR and HPLC-DAD analysis. The antioxidant activity, total phenolic content (TPC), and flavonoid content (TFC) were found to be higher in the ethanolic extracts, as compared to the aqueous extracts. From the above study, it can be demonstrated that *Urtica dioica* showed the highest antioxidant activity. Additionally, an in vitro examination stated that all of the mentioned medicinal plants induced programmed cell death in the HepG2 cells. IR illustrated the highest anticancer potential, trailed by UD, and CBP showed the least cytotoxicity activity after 48 h. Therefore, these herbs could be a source of conventional antioxidants and an impending hepatocellular cancer treatment. In the future, different biological activities can be determined to understand the effect of these infusions on a biological system.

## Figures and Tables

**Table 1:** Illustration of the correlation coefficient (R^2^-value) between the antioxidant ability through DPPH and ABTS assays with the TPC and TFC of aqueous (AQ) and ethanolic (ETH) fractions of *Urtica dioica* (UD), *Capsella bursa-pastoris* (CBP) and *Inula racemosa* (IR).

Samples	R^2^-Value DPPH/ABTS-TPC	Correlation Association	R^2^-Value DPPH/ABTS-TFC	Correlation Association
UD (AQ)	0.919 and 0.853	Strongly correlated	0.842 and 0.758	Strongly correlated
UD (ETH)	0.959 and 0.941	Strongly correlated	0.892 and 0.864	Strongly correlated
CBP (AQ)	0.994 and 0.929	Strongly correlated	0.982 and 0.951	Strongly correlated
CBP (ETH)	0.935 and 0.992	Strongly correlated	0.906 and 0.980	Strongly correlated
IR (AQ)	0.974 and 0.990	Strongly correlated	0.677 and 0.413	Moderately correlated
IR (ETH)	0.879 and 0.749	Strongly correlated	0.950 and 0.997	Strongly correlated

**Table 2:** FT-IR spectral data of the ethanolic extract of *U. dioica*.

S. NO	Peak Number (cm^−1^)	Bond Type	Functional Group
1	3329	OH Stretching	Alcohols
2	2922	C-H stretching	Alkanes
3	2858	C-H stretching	Aldehyde
4	1619	C=C stretch	Aromatic compounds
5	1399	C-H bending	Methylene group
6	1048	C-O stretch	Aliphatic amines
7	714	C-Cl stretch	Alkyl and aryl halides

**Table 3:** FT-IR spectral data of the ethanolic extract of *Capsella bursa-pastoris*.

S. NO	Peak Number (cm^−1^)	Bond Type	Functional Group
1	3289	OH Stretching	Phenols and alcohols
2	2922	C-H stretching	Alkanes
3	1608	C=C stretching	Aromatic compounds
4	1512	N-O stretching	Nitro compounds
5	1358	C-H bending	Methylene group
6	1045	C-O stretch	Aliphatic amines
7	540	C-I stretch	Alkyl and aryl halides

**Table 4:** FT-IR spectral data of the ethanolic extract of *Inula racemosa*.

S. NO	Peak Number (cm^−1^)	Bond Type	Functional Group
1	3349	O-H Stretching	Aliphatic primary amine
2	2924	C-H stretch	Alkanes
3	2859	C-H stretch	Alkene
4	1741	C=O stretch	Ketones
5	1655	C=C Stretch	Conjugated alkane
6	1455	C-H bending	Methylene group
7	1369	NO_2_ stretch	Nitro compounds
8	1259	C-F stretch	Alkyl and aryl halides
9	1038	C-F stretch	Alkyl and aryl halides
10	812	C-Cl stretch	Alkyl and aryl halides
11	658	C=C bending	Alkene
12	660	C=C bending	Alkene
13	618	C-Br stretch	Alkyl and aryl halides

## Data Availability

Data are contained within the article.

## Abbreviations

*Urtica dioica* (UD), *Capsella bursa-pastoris* (CBP), *Inula racemosa* (IR), 2,2-diphenylpicrylhydrazyl (DPPH), 2,2′-azino-bis-3-ethylbenzothiazoline-6-sulfonic acid (ABTS), Attenuated total reflection-Fourier transform infrared spectroscopy (ATR-FTIR), High-performance liquid chromatography-Dioide array detector (HPLC-DAD), Gas chromatography-Mass spectroscopy (GC-MS), Liver hepatocellular carcinoma (HepG2), Total phenolic content (TPC), Total flavonoid content (TFC), Reactive oxygen species (ROS), Nuclear erythroid 2-related factor 2-Antioxidant response element (Nrf2-ARE), Heme oxygenase-1 (HO-1), Glutamate cysteine ligase (GCL), Mallard reaction product (MRP), Kelch-like associating protein-1 (KEAP-1), Alanine aminotransferase (ALT), Aspartate aminotransferase (AST), Lactate dehydrogenase (LDH), Optical density (OD), Aqueous (AQ), Ethanolic (ETH), Dimethyl sulfoxide (DMSO), Effective concentration (EC_50_), Gallic acid equivalents (GAE), Quercetin equivalents (QE).
